# Acute Ileosigmoid Knotting in an Adult: CT Diagnosis of a Rapidly Progressive Closed-Loop Volvulus

**DOI:** 10.7759/cureus.107210

**Published:** 2026-04-17

**Authors:** Ínigo Fuentes Bermejo, Pir Abdul Ahad Aziz Qureshi, Emil Ólason, Hulda María Einarsdóttir, Enrico Arkink

**Affiliations:** 1 Department of Radiology, Landspítali - The National University Hospital of Iceland, Reykjavík, ISL; 2 Faculty of Medicine, Háskóli Íslands, Reykjavík, ISL; 3 Department of Surgery, Landspítali - The National University Hospital of Iceland, Reykjavík, ISL

**Keywords:** bowel ischemia, closed-loop obstruction, compound volvulus, ct, ileosigmoid knot, midgut volvulus

## Abstract

Ileosigmoid knotting (ISK) is a rare form of compound volvulus in which ileal loops wrap around the sigmoid colon or vice versa, producing a rapidly progressive closed-loop obstruction with a high risk of ischemia. Prompt computed tomography (CT) recognition is essential, as clinical findings are often non-specific, and deterioration can occur quickly.

We report the case of a previously healthy adult who presented with abrupt, severe abdominal pain and progressive distension. CT revealed a double closed-loop configuration involving the distal ileum and sigmoid colon, a prominent whirlpool sign, reduced mural enhancement of ileal loops, mesenteric congestion, and localized free fluid. Emergency laparotomy confirmed ISK, with non-viable ileal segments requiring resection. The postoperative course was uneventful.

This case highlights the critical role of CT in diagnosing ISK and identifying early signs of ischemia. Systematic assessment of anatomy, loop orientation, and enhancement characteristics is essential to expedite surgical management.

## Introduction

Ileosigmoid knotting (ISK), also referred to as compound volvulus, represents a rare, yet life-threatening cause of acute abdomen that arises from the simultaneous twisting of ileal loops and the sigmoid colon volvulus [1,2]. The resulting interlocking loops form a double closed-loop obstruction, which quickly impairs venous outflow and, subsequently, arterial perfusion, ultimately leading to gangrene if not promptly treated [1,3]. The clinical presentation often mimics more common etiologies of bowel obstruction, contributing to diagnostic delays and increased mortality [2].

Computed tomography (CT) is the preferred imaging modality for early detection. Characteristic findings include the whirlpool sign (twisting of mesenteric vessels and bowel loops), crowding and twisting of both small bowel and sigmoid mesentery, abnormal enhancement, and abrupt transition points [1,3]. Accurate preoperative identification substantially improves patient outcomes, as timely surgical intervention is critical [4].

This report presents a case of ISK in an adult patient, emphasizing key CT features, diagnostic challenges, and correlation with operative findings.

## Case presentation

A previously healthy female presented to the Emergency Department with sudden-onset severe abdominal pain that developed over several hours, accompanied by nausea, vomiting, and progressive abdominal distension. On examination, the abdomen was distended and diffusely tender, with guarding. Laboratory studies demonstrated leukocytosis and mild metabolic acidosis (Table 1).

**Table 1:** Blood investigation results of the patient at the time of admission TLC: total leukocyte count; Hg: hemoglobin; CRP: C-reactive protein

Investigations	Values on admission	Reference range
TLC	28.1 x 10^9^/L	4.0-10.5 x 10^9^/L
Neutrophils	23.7 x 10^9^/L	1.9-7.0 x 10^9^/L
Hg	17.5 g/dL	11.8-15.2 g/dL
CRP	<3 mg/L	<10 mg/L
Creatinine	101 µmol/L	50-90 µmol/L
Blood glucose	11.7 mmol/L	4-6 mmol/L
Arterial blood pH	7.32	7.35-7.45

**Figure 1:**
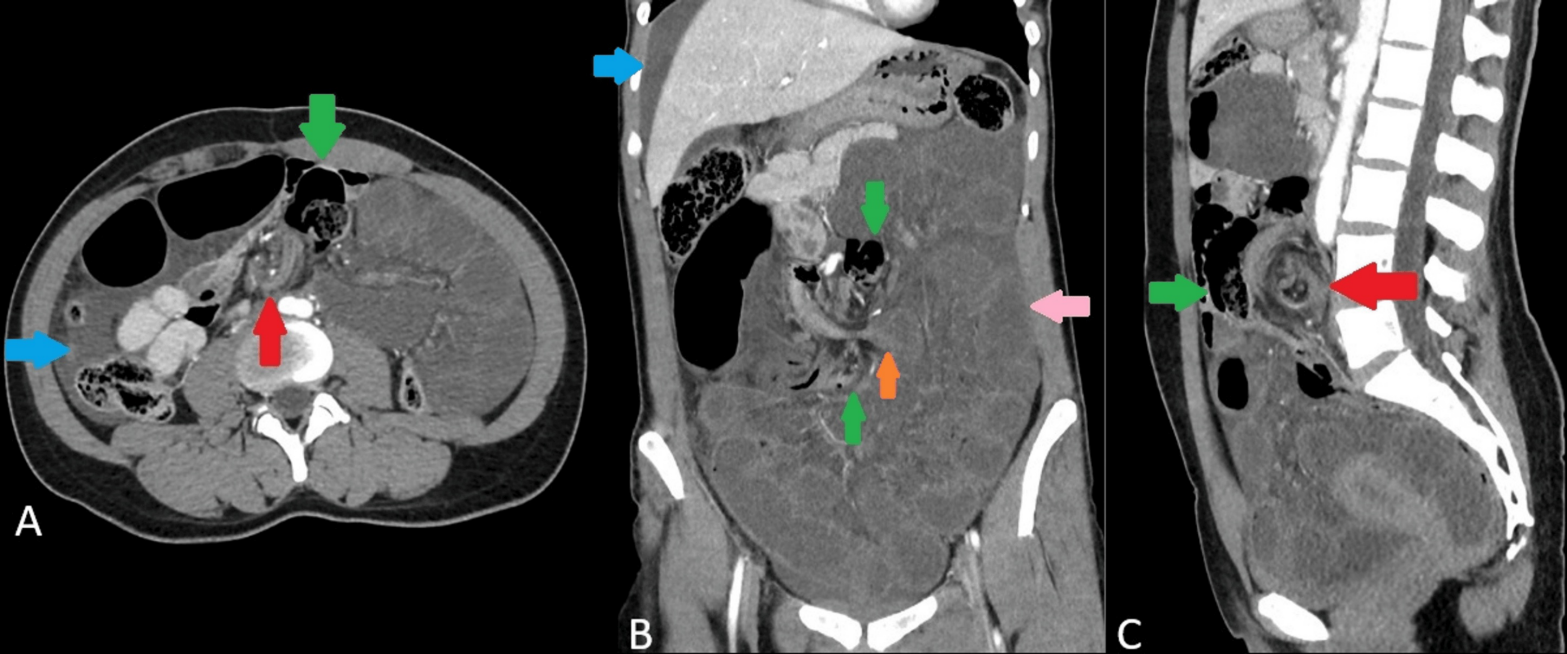
CT scan abdomen with contrast (axial, coronal and sagittal views) Contrast-enhanced CT abdomen axial view (A), coronal view (B), and sagittal view (C) showing twisting of the mesentery, giving a whirlpool pattern (red arrows in A and C), with resultant dilatation of the small bowel loops (orange arrow in B), which also show decreased enhancement, suggesting ischemia (pink arrow in B). Notice the sigmoid colon, which is also displaced superiorly and rotated around the mesenteric axis (green arrows in A, B, and C), and ascites (blue arrow in A and B). No pneumatosis, portal venous gas, or pneumoperitoneum is seen. CT: computed tomography

Due to concern for acute bowel obstruction and possible ischemia, an urgent contrast-enhanced CT of the abdomen and pelvis was obtained. Contrast-enhanced CT revealed markedly dilated distal ileal loops forming a compact closed-loop in the central abdomen. The superior mesenteric artery and vein branches were twisted in a whirlpool pattern, with prominent venous congestion. Arterial opacification was reduced in the affected segments but remained visible proximally, suggesting partial compromise of arterial flow. The involved ileal segments showed decreased mural enhancement, consistent with ischemia. The mesentery was edematous, with multiple engorged draining veins. Moderate reactive free fluid was present in the pelvis and perihepatic space. The sigmoid colon was involved in the torsion, displaced superiorly, and rotated around the mesenteric axis. Although distended, its wall enhancement was relatively preserved. No pneumatosis, portal venous gas, or pneumoperitoneum was identified. Other abdominal organs were unremarkable. The findings were promptly communicated to the surgical team, and an emergency exploratory laparotomy was performed (Figure 1).

**Figure 2:**
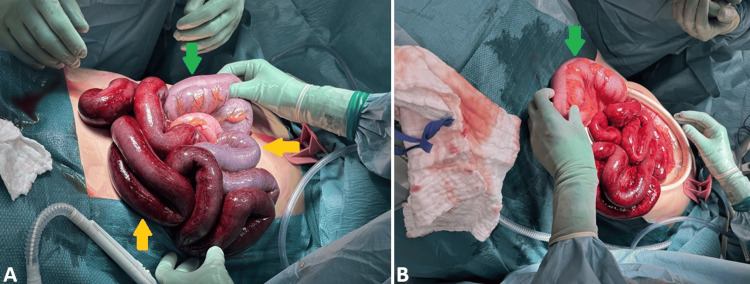
Intraoperative image showing ileosigmoid knotting (A) Extensive ischemia of the small bowel loops (yellow arrows) and the volvulized sigmoid colon (green arrow) is evident, with darkened loops and absent peristalsis. (B) Ileosigmoid knotting is demonstrated by small bowel loops wrapped around a twisted sigmoid colon (green arrow). The sigmoid colon was volvulized, acting as the fixed axis around which the ileum had rotated.

Intraoperatively, extensive small bowel ischemia was identified, but no perforation. The small intestine was dark and congested, extending proximally from a clear demarcation at the ileocecal junction. A long, redundant sigmoid colon with a narrow mesenteric base had undergone volvulus, and the distal small bowel was tightly knotted around the volvulized sigmoid colon, confirming ISK. Despite complete detorsion, the affected small bowel remained non-viable, with absent pulsation and persistent discoloration. Resection of the ischemic distal small bowel was performed, leaving approximately 140 cm of viable proximal small intestine from the ligament of Treitz. Due to the volvulized configuration and high risk of recurrence, a sigmoid colectomy was also performed, followed by a primary isoperistaltic side-to-side colocolonic anastomosis. A side-to-side anastomosis was created between the remaining small bowel and cecum. The appendix was removed prophylactically due to the altered anatomy (Figure 2).

**Figure 3:**
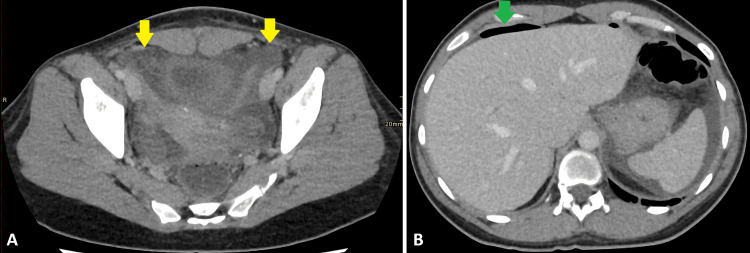
CT scan abdomen with contrast (axial views) Contrast-enhanced CT abdomen, axial views (A and B), showing expected postoperative changes in the form of free fluid (yellow arrows in A) and pneumoperitoneum (green arrow in B). CT: computed tomography

The patient stabilized postoperatively. A CT scan was performed on the fourth postoperative day for persistent abdominal pain. This revealed expected postoperative changes, without evidence of anastomotic leak or intra-abdominal collection. Bowel function gradually returned, and the patient was discharged with routine surgical follow-up (Figure 3).

Histopathological examination of the resected distal small bowel confirmed extensive ischemic injury, with predominant mucosal necrosis, focal full-thickness necrosis, and associated hemorrhage. Ischemic changes were present at both resection margins but were less pronounced than in the central segments. The resected sigmoid colon showed mucosal ischemic necrosis, without extension into the deeper layers of the bowel wall. Resection margins were viable. The appendix was histologically unremarkable. These findings were consistent with severe ischemic injury secondary to closed-loop obstruction.

## Discussion

ISK represents a distinctive subtype of compound volvulus in which the ileum and sigmoid colon become mutually twisted, producing a fulminant closed-loop obstruction [1]. Mortality remains high, historically reported between 20% and 50%, largely due to delayed diagnosis and rapid progression to gangrene [2,3]. Multiple anatomical and physiological factors have been implicated, including a long sigmoid mesocolon, hypermobile small bowel, and dietary triggers [2,5].

CT plays a decisive role in early and accurate diagnosis of ISK and is the imaging standard for preoperative recognition [1-4]. The whirlpool sign, characterized by spiraling of mesenteric vessels and fat around a central torsion point, is one of the most reliable imaging features and is typically present in multiple planes [1-3]. Another hallmark is identification of a double closed-loop configuration, in which both the distal ileum and the sigmoid colon are simultaneously involved, which helps distinguish ISK from isolated sigmoid volvulus or midgut volvulus [1]. These findings help differentiate ISK from other causes of closed-loop obstruction, such as adhesions, internal hernia, or isolated sigmoid volvulus. CT is also essential for evaluating the degree of ischemia. Reduced mural enhancement, mesenteric edema, venous engorgement, free fluid, and wall thickening or thinning are well-described indicators of compromised perfusion. Early CT recognition is crucial because it enables immediate surgical consultation and reduces the risk of progression to bowel gangrene [4].

The anatomical classification of ISK is based on identifying the segment of bowel that functions as the active component initiating the wrapping, and the passive axis. Type I ISK, the most common form, accounts for approximately 54% to 58% of cases and involves the ileum acting as the active component, wrapping around the base of the sigmoid colon. In contrast, Type II occurs in 19% to 21% of cases, with the redundant sigmoid colon serving as the active driver and encircling a stationary loop of ileum. Type III is a rare variant, representing about 1.5% of cases, in which the ileocecal segment wraps around the sigmoid colon. Both Type I and Type II are further divided into subtypes A and B, depending on whether the torsion is clockwise or counterclockwise. In advanced cases with extensive gangrene or severe distension, anatomical distortion may prevent differentiation of the active and passive components, resulting in a Type IV or undetermined classification [6,7]. This is further summarized in Table 2.

**Table 2:** Anatomical classification of ileosigmoid knotting based on identification of active and passive bowel segments and their relative frequencies Source: [6,7]

Type	Active component	Passive component	Frequency (%)
Type I	Ileum	Sigmoid Colon	53.9-57.5%
Type II	Sigmoid Colon	Ileum	18.9-20.6%
Type III	Ileocecal Segment	Sigmoid Colon	~1.5%
Type IV	Undetermined	Undetermined	Rare

Despite its characteristic features, ISK can be challenging to diagnose radiologically. In early or partial torsion, the whirlpool sign may be subtle or visible only in certain reconstruction planes [1,3]. Initial CT may demonstrate nonspecific bowel dilatation and mesenteric congestion without definitive evidence of a dual-loop configuration, leading to potential underdiagnosis [2]. Closed-loop obstructions caused by adhesions or internal hernias may closely mimic ISK, particularly when mural enhancement is preserved [1]. Another challenge arises when the sigmoid colon is decompressed; its involvement may be missed unless carefully traced through multiplanar reformats [3]. Because ischemia can progress rapidly between imaging and intervention, radiologic assessment must always be correlated closely with clinical deterioration [2-4]. Careful evaluation of mesenteric orientation, vascular rotation, and transition points is essential to avoid misinterpretation.

Management is exclusively surgical, with emergency laparotomy indicated in all confirmed cases. Delay significantly increases the likelihood of gangrene due to the double closed-loop mechanism [1,2]. Reported literature consistently supports resection of non-viable ileal segments, with sigmoid resection depending on perfusion status [3,4].

ISK is associated with a high mortality rate, reported to reach up to 48%. However, some studies have reported lower rates, such as a six-year review from Ethiopia that reported a mortality rate of 7.9% [8,9]. Higher mortality is linked to factors such as older age, pregnancy, existing comorbidities, and delays in presentation and diagnosis [8].

Our case demonstrates the typical epidemiological and anatomical patterns of ISK. The patient, a young adult, fits the age group where this condition is most common, usually affecting adults in their 30s to 50s. During surgery, we found a Type I knot, where the ileum wraps around the sigmoid colon. This is the most common form of the disease. Furthermore, this case also illustrates the characteristic imaging constellation of ISK and reinforces the value of systematic analysis of mesenteric vasculature, loop configuration, and enhancement patterns in patients with acute abdomen. Given its rarity, heightened awareness is important for radiologists, particularly in emergency settings.

## Conclusions

ISK is a rare yet life-threatening cause of acute abdomen that can progress rapidly to bowel ischemia, resulting in significant morbidity and mortality if not identified promptly. Due to the nonspecific clinical presentation and overlapping imaging findings, preoperative diagnosis remains challenging, often delaying management. In this context, CT plays an essential role in early diagnosis, identification of ischemic changes, and guidance of surgical planning. Increased awareness among clinicians and radiologists, together with timely imaging and decisive surgical management, can substantially improve patient outcomes. Additionally, further case reports and larger studies are needed to better characterize early imaging features and refine diagnostic strategies for the timely recognition of this rapidly progressive condition.
